# Plasma Treatment Enhances Cytocompatibility on Patterned Perfluoroethylene Propylene

**DOI:** 10.3390/jfb17080364

**Published:** 2026-07-28

**Authors:** Bára Frýdlová, Nikola Slepičková Kasálková, Irena Vacková, Šimon Pražák, Lucie Bačáková, Zdeňka Kolská, Esther Rebollar, Václav Švorčík, Petr Slepička

**Affiliations:** 1 Department of Solid State Engineering, The University of Chemistry and Technology Prague, 6-Dejvice, 166 28 Prague, Czech Republic; bara.frydlova@vscht.cz (B.F.); nikola.kasalkova@vscht.cz (N.S.K.); vaclav.svorcik@vscht.cz (V.Š.); 2 Institute of Physiology of the Czech Academy of Sciences, Vídeňská 1083, 4-Krč, 142 00 Prague, Czech Republiclucie.bacakova@fgu.cas.cz (L.B.); 3 Faculty of Science, Jan Evangelista Purkyně University in Ústí nad Labem, Pasteurova 3544/1, 400 96 Ústí nad Labem, Czech Republic; 4 Instituto de Química Física Blas Cabrera, CSIC, C. de Serrano 119, 28006 Madrid, Spain

**Keywords:** replication, microstructure, nanostructure, polymer, plasma activation, surface morphology, FEP, cytocompatibility, mesenchymal stem cells

## Abstract

This study aims to construct patterns with defined shapes on perfluoroethylene propylene (FEP) via a replication process that has potential applications in tissue engineering. Our focus was on creating ripple periodic submicron-scale structures and subsequently activating them with argon plasma. Plasma activation of the replicated pattern on FEP substrates proved to be an effective, easy-to-execute and cost-efficient treatment method. Structured and activated carriers were prepared to study targeted cell growth in terms of cell adhesion and proliferation. Furthermore, the carriers were used to influence cell shape and the direction of the cell growth based on the properties of the pattern, primarily its morphology and dimensions. The carriers were seeded with human Wharton’s jelly stromal cells and their metabolic activity and morphology were studied using a resazurin assay and fluorescence staining of the actin cytoskeleton and cell nuclei. We found that plasma treatment improves cell adhesion of the material more effectively than patterning the material with submicron-scale grooves (approximately 500 nm wide). However, this patterning enhances the susceptibility of the material to plasma modification, as evidenced by the subsequent improvement in cell growth.

## 1. Introduction

The area of nanostructured polymer film surfaces has significantly increased in importance during the last decades. The fabrication of nano-engineered surfaces to obtain micro- and nano-patterns [1] has been achieved using several advanced techniques. Strategies for pattern construction can be generally divided into bottom-up and top-down approaches [2]. The bottom-up approach is based on the idea that structure formation is achieved using small building blocks with self-assembling properties. Typical examples of these materials include amphiphilic lipids, surfactants, block copolymers, and colloidal particles. The second approach, called the top-down approach, is based on the idea that originally homogeneous materials are shaped by techniques such as lithography, or that layers are created by vacuum evaporation or sputtering from a bulk source [3]. The most frequently used approaches, in general, may also be divided into the following major groups, frequently used for top-down approaches: techniques, such as moulding (hot embossing) (i), techniques based on nano/microimprinting or techniques based on laser ablation (ii), construction of nanopatterns with soft lithography (iii) and last but not least, (iv) simple laser scanning mostly used for periodic pattern construction in case of polymer surfaces, where the pattern, such as ripples or wrinkles, can be constructed due to surface instability and absorption of energy [4,5,6]. Surface instabilities during pattern formation may lead to a pattern that is not strictly periodic and is described as wrinkles, or a wrinkle-like pattern, which can be prepared either by means of polymer casting, imprinting, or dewetting, or also in combination with other techniques, such as plasma exposure and consecutive heating [7,8,9,10,11,12].

Some approaches to the construction of surface-patterned polymers have been intensively studied over the past decade. The recent interest is based mostly on the possibility of preparing complex patterns through self-organizing processes, which are more difficult than fabricating them using traditional patterning techniques. One of the most promising techniques for preparing patterned surfaces/interfaces is the formation of wrinkle-like patterns [4,5,6]. Fabrication approaches aimed at inducing wrinkle formation and precisely controlling surface roughness, morphology, the period and height of the wrinkles, and their functionality and final morphology are intensively studied [2,6].

Surface instabilities in polymer films occur primarily when active interactions between polymer interfaces are present. Instability can also be introduced in the “stable system” by the application of external forces. It has been shown previously that surface instability arises from the competition between destabilizing and stabilizing forces [7]. The present forces may be described as Van der Waals forces, steric interactions, or electrostatic interactions; if external forces are applied to the system, surface tension or elastic strain may be present [7]. The presence of such forces results basically into two different situations: (i) appearance of an unstable film as a consequence of the inherent dynamics, which may lead, e.g., to dewetting [8,9], (ii) appearance of metastable films which become unstable under an external stimulus, e.g., temperature [10,11,12]. Instability in the latter may arise from, for instance, thermal gradients, mechanical forces, or an external electric field; typically, this type of instability (most frequently driven by an external heat stimulus) may lead to the formation of a wrinkle pattern. The surface instability results in an evolution of the film structure, considering a “more optimal” situation in terms of surface energy. Therefore, an appropriate understanding and control of the parameters that influence surface pattern, based on surface instability, is a very simple yet interesting alternative for constructing innovative patterns on solid-state substrates with precise control of surface morphology [12].

As stated above, several types of surface instabilities can be employed to create surface morphologies with varying dimensions or shapes. Dewetting, which may be described as the rupture of the film, forming random morphologies, is guided by the free energy of the system [8,13]. By this process, surfaces with random distribution of holes, multilayer structures, or polygons were observed, and the process may also be used for patterning polymeric materials using heterogeneous substrates [14]. Typically, this process can produce a large variety of surface patterns, for instance, 2D ordered arrays of polymer ring structures [15]. Considering other methods, the most used are phase separation of polymer blends and block copolymers [16,17], template guided structuration [18], electrohydrodynamic patterning [19] or thermal-gradient induced surface patterning, where an externally imposed temperature gradient causes a surface rippling with a characteristic wavelength that finally leads to the film modification into different morphologies [20], temperature gradient driven instability produces replication patterns [21]. Surface wrinkles, creases, and even folds are common instability phenomena in polymer films [22,23]. This phenomenon is based either on mechanical stress (e.g., osmotic pressure, stretching) or on heating, where a rigid layer is deposited on top of a polymer film. If the applied stress is removed, the film relaxes, and a wavy structure, generally known as “wrinkles,” forms. The use of these materials as templates for flexible electronics, in combination with detailed control of substrate wettability and/or cell adhesion, is being intensively studied. Such interfaces have already been employed in electronics, tissue engineering, and cell growth control, and may serve as a basis for constructing polymer-based substrates for detecting chemicals or elements [24,25,26,27,28].

In this study, we applied patterning with wavy, wrinkle-like structures to perfluoroethylene propylene, also known as fluorinated ethylene propylene (FEP), a polymer similar to polytetrafluoroethylene (PTFE, Teflon^®^), but which can be more easily processed, welded, and molded into complex shapes. Due to its excellent mechanical, thermal, optical, electrical, and chemical properties, FEP shows promise in a wide range of industrial and biomedical applications, including cell therapy and tissue engineering [29,30,31]. In this study, we explored this potential application with mesenchymal stem cells derived from Wharton’s jelly of the human umbilical cord. This cell type is relevant to various fields of tissue engineering because it can be differentiated into multiple phenotypes—not only osteoblasts, chondrocytes, and adipocytes, as is typical for mesenchymal stem cells [31,32], but also towards vascular endothelial and cardiac muscle cells, as well as various ectodermal and endodermal cell types.

## 2. Materials and Methods

### 2.1. Materials

Polymer polydimethylsiloxane (PDMS), in the form of an elastomer kit (Sylgard 184^®^; base/curing agent ratio 10:1), was purchased from Sigma-Aldrich (Merck, Darmstadt, Germany) and used in the experiments. The commercially available digital versatile disc-recordable (DVD-R) (Verbatim GmbH, Eschborn, Germany; 4.7 GB) was purchased from the market. As a primary substrate for hot embossing, we used FEP polymer (density 2.15 g·cm^−3^; 50 μm-thick foils) supplied by Goodfellow Ltd., Great Britain. This foil was cut into different sizes (approx. 1 cm^2^) depending on the particular analysis, or discs 20 mm in diameter for cell experiments. Similarly, plasma-treated FEP was exposed to plasma (8 W, 240 s), typically 1 cm^2^ for analyses such as atomic force microscopy (AFM) or scanning electron microscopy (SEM), or 20 mm-diameter discs for cell experiments.

### 2.2. Plasma Treatment

Surface activation was carried out using a diode argon plasma discharge generated in a Balzers SCD 050 system (Balzers, Liechtenstein). Argon with a purity of 99.997% was introduced at a pressure of 10 Pa. The plasma treatment was performed at room temperature using discharge powers of either 3 or 8 W, with the electrode-to-sample distance fixed at 60 mm. The exposure time was varied from 0 to 240 s to evaluate the effect of plasma duration on the resulting surface properties.

### 2.3. Replication

The patterned polycarbonate (PC) layers of commercially available DVD-R discs (4.7 GB; Verbatim GmbH, Eschborn, Germany) were employed as master templates. Circular sections with a diameter of 50 mm were cut from the discs, after which the individual PC layers were separated. A DVD-R disc is composed of two polycarbonate substrates with a reflective layer and a dye-containing layer positioned between them. The two PC components were manually detached along the cut edge using tweezers. The grooved PC layer was subsequently immersed in methanol and sonicated for 30 min to remove residual reflective material, dye, and surface contaminants such as dust particles. The substrate was then repeatedly rinsed with distilled water and methanol, yielding a clean DVD-R master suitable for the replication procedure.

#### 2.3.1. Fabrication of PDMS Molds

PDMS was used to replicate the surface structure of DVD discs using the soft lithography (cast molding) technique. The PDMS base and curing agent were thoroughly mixed by hand at a 10:1 weight ratio and then poured over PC master molds placed in Petri dishes. After subsequent degassing in a vacuum chamber for 1 h, the Petri dishes were thermally annealed in an oven at 80 °C for 2 h to complete polymer cross-linking. Once the thermoset was cured, the flexible negative mold was carefully peeled off the PC master.

#### 2.3.2. Hot Embossing

The preformed FEP film was placed on the PDMS mold and covered by an additional layer of Teflon foil. The mold was then placed in a specially crafted Dural clamp, compressed under high pressure, and the whole system was put in a Binder oven at 300 °C and thermally annealed for 30 min. Teflon foil was added as a protective layer because it withstands high temperatures and prevents the melted polymer from sticking to the metal clamp. After cooling at room temperature (25 °C), the clamp pressure was released, and the FEP film was manually peeled off the flexible mold. Temperatures are reported as temperature intervals because the degree of crystallinity of this semi-crystalline thermoplastic determines its thermal properties.

### 2.4. Analytical Methods

#### 2.4.1. Atomic Force Microscopy Analysis

The surface topography and roughness of both untreated and modified films were characterized by atomic force microscopy (AFM) using a Dimension ICON instrument (Bruker Corp., Billerica, MA, USA). Measurements were performed in ScanAsyst^®^ mode with a SCANASYST-AIR nitride cantilever equipped with a silicon tip and a nominal spring constant of 0.4 N·m^−1^. The AFM data were evaluated using NanoScope Analysis software (version 1.80). The arithmetic mean of the roughness, Ra, was calculated as the mean of the absolute height deviations from the mean surface plane. Surface Area (S) is the three-dimensional area of a given region. This value represents the sum of the areas of all of the triangles formed by three adjacent data points. It is considered the integration of these areas within the real measured surface. Surface Area diff (in %) is the percentage increase of the three-dimensional surface area over the two-dimensional surface area.

#### 2.4.2. Scanning Electron Microscopy and EDS Analysis

The morphology of the sample surfaces was also characterized using a FIB-SEM LYRA3 GMU scanning electron microscope (Tescan, Brno, Czech Republic). The acceleration voltage was set to 10 kV. The elemental composition was measured by energy-dispersive X-ray spectroscopy (EDS; analyzer X-ManN, 20 mm^2^ SDD detector, Oxford Instruments, Abingdon, UK), with an accelerating voltage of 10 kV for SEM-EDS analysis.

#### 2.4.3. X-Ray Photoelectron Spectrometry

The elemental composition of the material surface was analyzed by X-ray photoelectron spectroscopy (XPS) using an ESCAProbeP spectrometer (Omicron Nanotechnology Ltd., Taunusstein, Germany). As a source, a monochromatic X-ray at an energy of 1486.7 eV was used. Atomic concentrations of elements were determined based on the areas of individual peaks using CasaXPS software 2.3.17PR 1.1.

#### 2.4.4. Wettability

The wettability of the investigated samples was assessed by contact-angle measurements (CA, θ) using a goniometer from Advex Instruments (Brno, Czech Republic) coupled with SEE System 7.1 software. All measurements were conducted at room temperature with 8 μL droplets of distilled water stained with methyl violet and dispensed using a Transferpette^®^ automatic pipette (Brand, Wertheim, Germany). Contact angles were recorded at six different locations on each of three samples, both parallel and perpendicular to the surface pattern. Images of the droplets were subsequently acquired, and contact angles were determined by fitting the droplet profile using three manually selected points.

#### 2.4.5. Zeta-Potential Determination

Electrokinetic analysis, more specifically, zeta potential determination of the tested films, was conducted using the SurPASS Instrument (Anton Paar GmbH; Graz, Austria) by two methods (streaming current and streaming potential) and calculated using the Helmholtz–Smoluchowski [HS] equation. The samples were studied in an adjustable-gap cell with 0.001 mol·dm^−3^ KCl as an electrolyte at a constant pH of 6.7 at room temperature.

### 2.5. Cytocompatibility Evaluation

#### 2.5.1. Cell Model and Culture Conditions

To evaluate material cytocompatibility, human Wharton’s jelly-derived mesenchymal stromal cells (hWJSCs), pooled from three donors, were used. The hWJSCs were prepared according to our previously established protocols [32]. Briefly, discarded human umbilical cords (UCs) were obtained from healthy full-term neonates (*n* = 3) after spontaneous delivery. Blood vessels were removed from the UCs, and the remaining Wharton’s jelly tissue (WJ) was chopped into small fragments. hWJSCs were isolated from fragments by enzymatic digestion with 0.26 U/mL liberase and 1 mg/mL hyaluronidase at 37 °C with constant shaking for 2 h. After the removal of undigested fragments with 40-μm cell strainers, cells were centrifuged, expanded in a complete culture medium (CCM) consisting of alpha-minimal essential medium (αMEM; Capricorn Scientific, Ebsdorfergrund, Germany, Cat. No. MEMA-RXA) supplemented with 5% pooled human platelet lysate (PL; Bioinova, Ltd., Prague, Czech Republic) and 10 μg/mL gentamicin (Sandoz, Holzkirchen, Germany), and then stored in liquid nitrogen. Cryopreserved cells at passage 3 were thawed and seeded into a CCM 3–5 days before the actual experiment into 75 cm^2^ tissue culture flasks (TPP Techno Plastic Products AG, Trasadingen, Switzerland) at a density of 5–6 thousand cells per cm^2^. After reaching 80–90% confluence, cells were harvested using trypsin-ethylenediaminetetraacetic acid (trypsin-EDTA, Sigma-Aldrich, Merck, Darmstadt, Germany, Cat. No. T4174), counted, and seeded onto FEP discs.

Human umbilical cords used for hWJSC derivation were obtained from the University Hospital in Pilsen (Czech Republic) from healthy newborns following spontaneous delivery. All umbilical cords were donated anonymously for research purposes, with written informed consent from the donors (mothers of newborns). The informed consents are stored at the University Hospital in Pilsen. All experiments involving human tissues or cells were approved by the Ethics Committee of the Institute of Physiology of the Czech Academy of Sciences in Prague.

#### 2.5.2. Cell Seeding

Before seeding, 2 cm diameter FEP discs were sterilized on both sides with 20 min of ultraviolet (UV) radiation (254 nm), transferred to a 12-well plate (TPP Techno Plastic Products AG, Trasadingen, Switzerland), and weighted down with glass rings of a diameter of 1.8 cm (Detesk Ltd., Železný Brod, Czech Republic) to anchor them to the bottom of the plastic wells. The tested discs were divided into 4 groups; see Table 1.

Each disc was seeded with 15,000 hWJSCs, i.e., at an initial cell population density of approximately 4777 cells per cm^2^. Tissue culture plastic (polystyrene) was used as a control, seeded with the same number of hWJSCs and cultured under the same conditions as the tested groups. The cells were cultured at 37 °C in a humidified air atmosphere containing 5% CO_2_ with regular media changes twice a week. Samples were tested in duplicates.

#### 2.5.3. Metabolic Activity of Cells

Metabolic activity of cells, considered a marker of cell viability and proliferation [33], was assessed using a resazurin metabolic assay on days 1, 3, and 6 after seeding hWJSCs on the tested materials, as described in our previous article [31]. However, as relatively immature cells with high proliferative capacity, the hWJSCs proliferated so rapidly that, between days 4 and 6, part of the cell layer detached in all samples; therefore, the cell metabolic activity on day 6 could not be included in the evaluation. Briefly, for the resazurin assay, the samples were transferred to new 12-well plates containing 2 mL of fresh CCM with 40 μM resazurin (Cat. No. R7017, Sigma-Aldrich, St. Louis, MO, USA) in each well and incubated at 37 °C in a humidified air atmosphere with 5% CO_2_ for 3.5 and 2 h for the first and third day of measurement, respectively. Then, 150 μL of the solution was added to 96-well plates, and the fluorescence (Ex/Em = 530/590) was measured on a Synergy^TM^ HT Multi-Mode Microplate reader (BioTek, Santa Clara, CA, USA). The measured values were corrected for the background control (CCM with resazurin). For each experimental group and time interval, two samples were used, and three measurements were performed on each sample (i.e., six measurements in total).

#### 2.5.4. Fluorescent Labeling of Cells

To visualize hWJSCs on FEP discs, filamentous actin (F-actin) in cells was stained with a phalloidin peptide conjugated to a fluorescent dye (Phalloidin-Atto 488; Cat. No. 49409, Sigma-Aldrich) on days 1 and 3 of culture. Briefly, cells were fixed with 4% paraformaldehyde and incubated in phosphate-buffered saline (PBS) containing 1% bovine serum albumin (BSA) and 0.1% Triton X-100 for 20 min to block nonspecific antibody binding sites and permeabilize the cell membrane. After PBS wash, the cells were incubated with phalloidin for 2 h. The cells were then washed twice with PBS (all from Sigma-Aldrich). The cell nuclei were counterstained with bisBenzimide H33258 (also known as Hoechst 33258; Cat. No. 94403; Sigma-Aldrich). Images were acquired on the Mateo FL Microscope (Leica Microsystems, Wetzlar, Germany). The images were used to evaluate cell morphology and the possible orientation of cells and their actin cytoskeleton relative to the DVD-like surface structure of the sample. The images were also used to directly count the cells adhering to and growing on the sample surface using ImageJ (v.1.54f; National Institutes of Health, Bethesda, MD, USA).

#### 2.5.5. Statistical Analysis

Statistical analysis of the quantitative cell-related data was performed using GraphPad Prism version 10.4.1 (GraphPad Software, San Diego, CA, USA). Data are expressed as mean ± SD. One-way ANOVA followed by the Student–Newman–Keuls post hoc test was used. A *p*-value ≤ 0.05 was considered statistically significant.

## 3. Results and Discussion

### 3.1. Atomic Force Microscopy of the FEP Samples

One of the key phenomena influencing the cytocompatibility of a material and the growth of cells with partial or full alignment ability is the surface morphology [34,35,36,37]. Therefore, we focused on constructing a submicron-grooved pattern from inert FEP. This polymer usually exhibits low cytocompatibility in its pristine state [38,39], but the additive plasma or laser treatment could substantially enhance its cytocompatibility. Based on our previous experiments involving the plasma treatment of either perfluorinated substrates [39] or polyolefins, the maximum applicable plasma power is 8 W. This combination was also used as the maximum activation power in our study, since higher plasma exposure power led to pronounced surface ablation of the polymeric material. Plasma-treated FEP samples have been described in detail in our previous studies [38,39]; therefore, these results will not be introduced explicitly in this paper.

**Figure 1:**
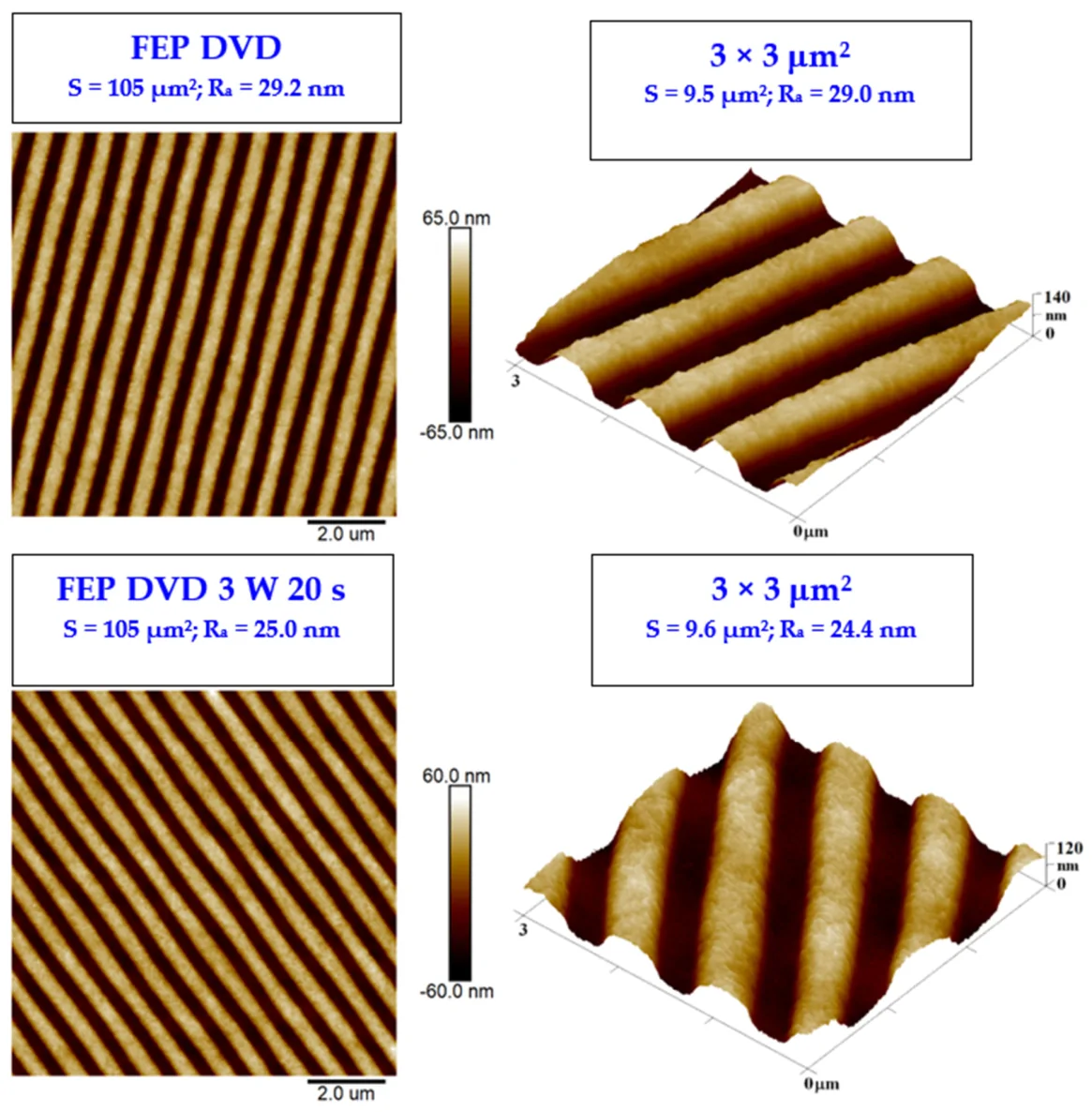
AFM images of DVD-replicated pristine FEP (the 1st line) and plasma-modified FEP foil with 3 W power and a plasma exposure time of 20 s (the 2nd line) are introduced. Squares of 10 × 10 μm^2^ (the 1st column) and 3 × 3 μm^2^ (the 2nd column) are shown. R_a_ represents the average surface roughness in nm, and S represents the effective surface area.

The surface morphology of plasma-treated material is visible in Figure 1. Our first goal was to confirm the successful replication process of the FEP foil and the maintenance of a regular linear pattern, which could support cell guidance. This was fully confirmed, as it is visible from the 1st line of Figure 1. It is evident that the linear pattern was successfully transferred into the FEP polymer. A detailed image also confirmed the quality of the replication process, showing a globular surface in detail, which will be discussed in the next paragraph. Plasma treatment at only 3 W could have a significant impact on the surface chemistry, but should not significantly affect the surface morphology. In our previous papers [38,39], we examined the effects of FEP plasma treatment on surface morphology, chemistry, and cytocompatibility. FEP can be enhanced by the ultraviolet and laser treatment [40]. We also applied an additive honeycomb layer to increase FEP cytocompatibility [41]. According to the results of these papers, the short treatment with lower plasma power did not significantly affect the surface. Detailed surface morphology also confirms the preservation of ripple pattern with insignificant structural changes; only slight changes in the effective surface roughness were observed during detailed scanning.

**Figure 2:**
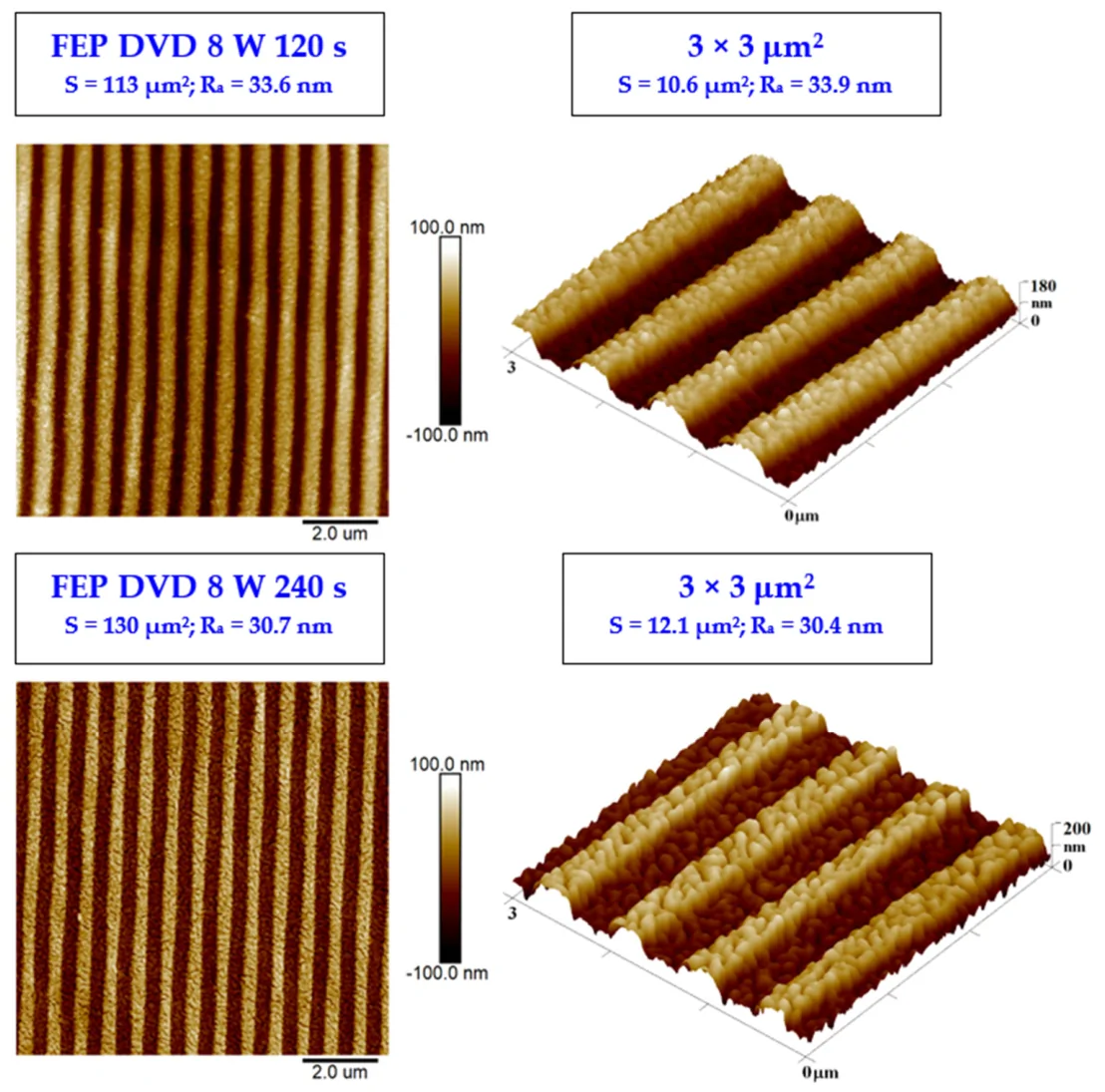
AFM images of DVD-replicated and plasma-modified FEP foils at 8 W power and plasma exposure times of 120 s and 240 s are presented. Squares of 10 × 10 μm^2^ (the 1st column) and 3 × 3 μm^2^ (the 2nd column) are shown. R_a_ represents the average surface roughness in nm, and S represents the effective surface area.

We have further investigated the effects of plasma treatment on a standard FEP pattern prepared via the replication process. For clarity, we present only the results for higher-power plasma (i.e., 8 W). Figure 2 shows the surface morphology of the resulting activated patterns for plasma exposure times of 120 and 240 s. Plasma treatment induced significant chemical changes to the FEP surface, which will be discussed in a later chapter. However, the primary morphological pattern remained periodic and linear with no disruption.

Examining the plasma-treated FEP with higher plasma power and higher exposure time (2nd line of Figure 2) in more detail reveals that the plasma activation significantly alters the detailed surface morphology. The wrinkle-like structure is superimposed on the linear, periodic, submicron-scale surface pattern of the replicated FEP. In combination with significant chemical changes, these factors are both significant for cell adhesion and growth and will be discussed in the last chapter of this paper. Comparing the effective surface area of 3 × 3 μm^2^ scans, we observe a notable increase from 9.5 μm^2^ to 10.6 μm^2^ for the combination of 8 W plasma and 120 s exposure time, and up to 12.1 μm^2^ for 240 s exposure time. This corresponds to an increase in the effective surface area by more than 30%. The increase of effective surface area is accompanied by an increase in surface roughness, from approximately 25 to values over 30 nm.

**Figure 3:**
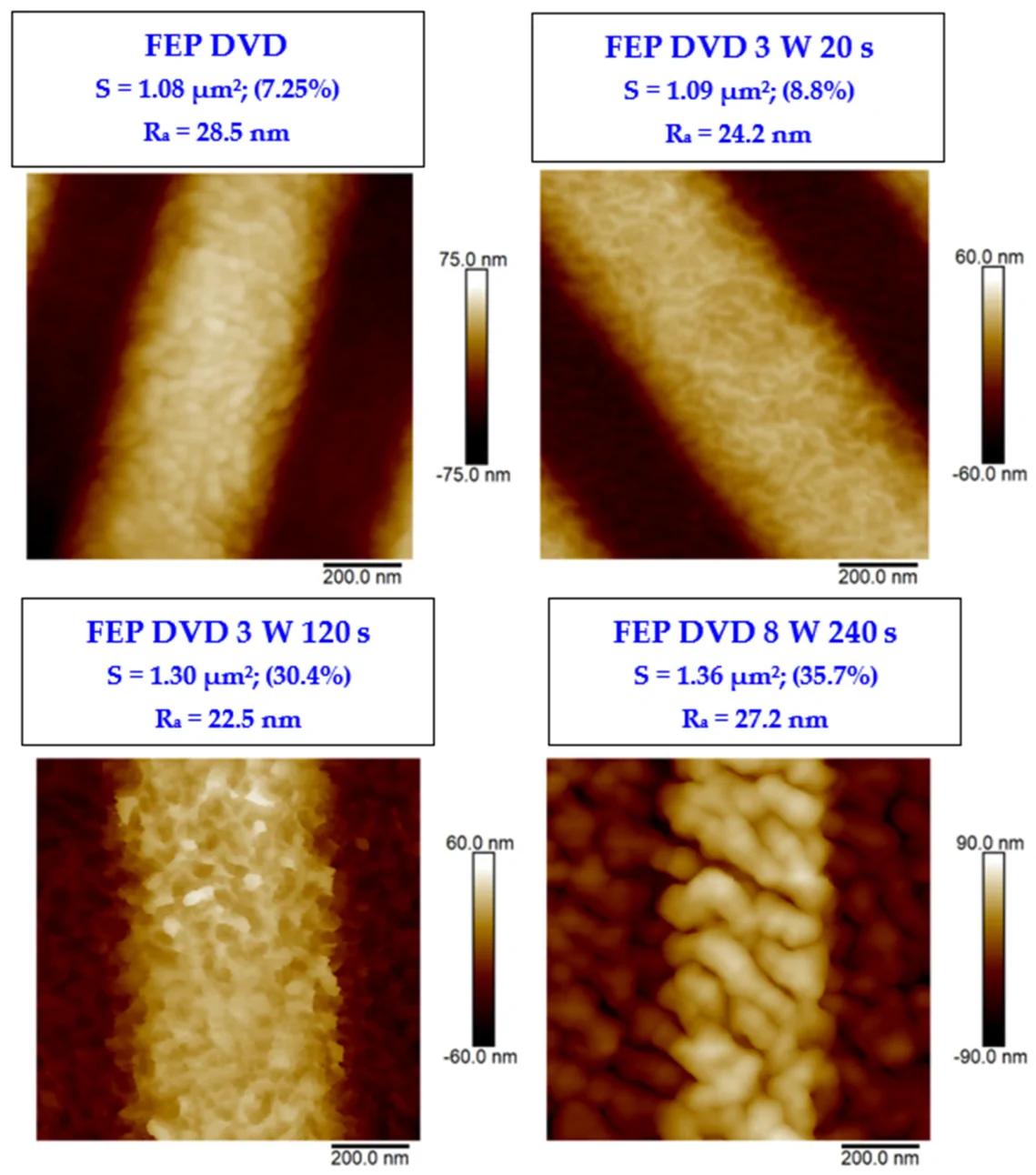
Detailed AFM images of DVD-replicated pristine FEP and plasma-modified replicated FEP foils at 3 W power (20 s and 120 s) and 8 W power (240 s) are presented. Squares of 1 × 1 μm^2^ are shown. R_a_ represents the average surface roughness in nm, S represents the effective surface area, and the number in % represents the difference between the basic planes.

The changes in surface morphology induced by plasma treatment are demonstrated in detail in Figure 3. As is obvious, although the linear structure is maintained after plasma treatment, this treatment induces a significant change. Higher plasma power and exposure time may result in a slight decrease in amplitude, as seen in the detailed images. Generally, these changes are induced by the ablation of the FEP polymer, as confirmed in our previous studies [38,39], and have a positive influence on filopodia attachment [42]. The cytocompatibility results will be presented further in this paper.

### 3.2. Scanning Electron Microscopy and Energy Dispersive Analysis

**Figure 4:**
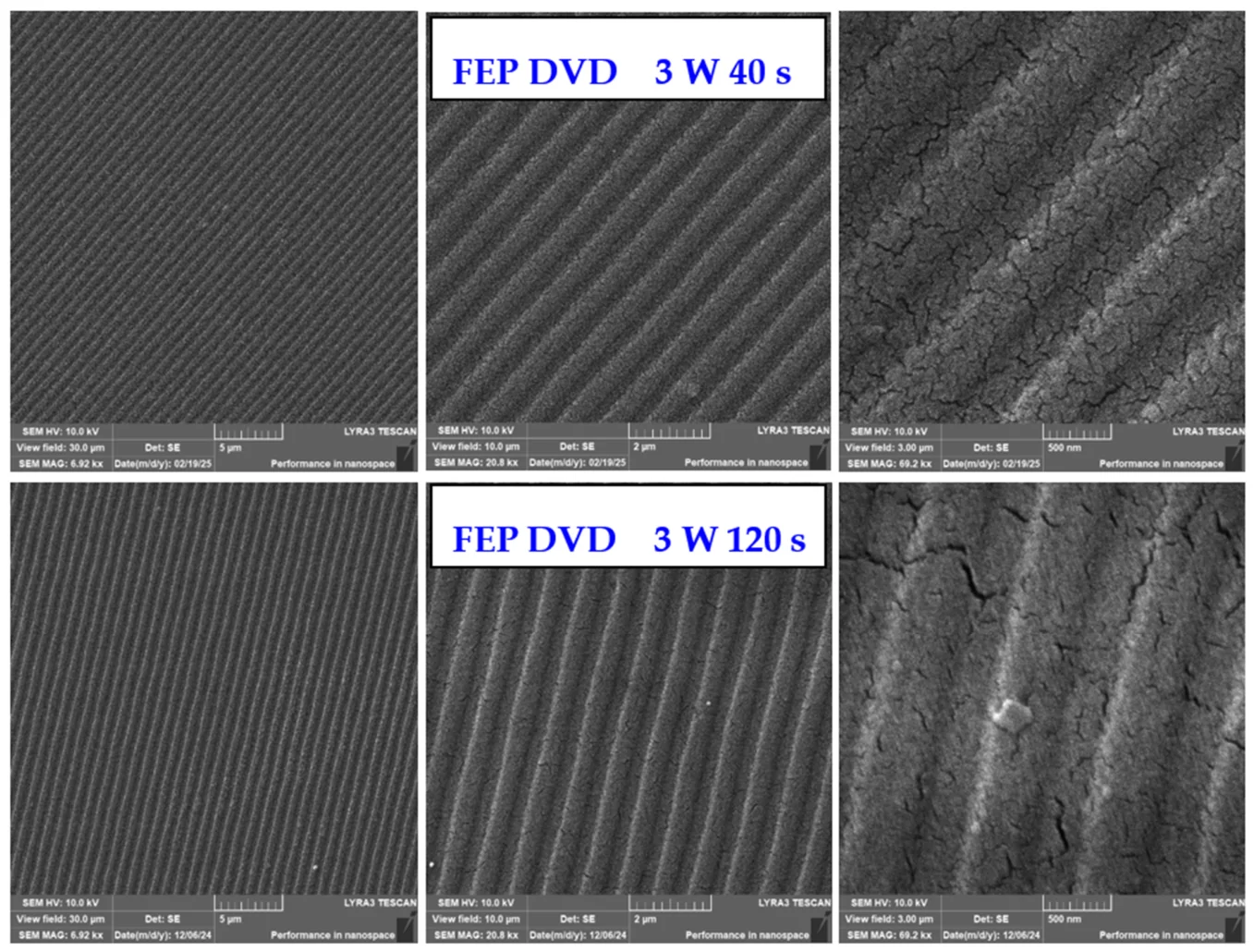
SEM images of DVD-replicated and plasma-modified FEP foils with 3 W power and plasma exposure times of 40 s and 120 s were chosen. Squares of 30 × 30 μm^2^ (the 1st column) 10 × 10 μm^2^ (the 2nd column) and 3 × 3 μm^2^ (the 3rd column) are shown.

The surface morphology was also examined using a scanning electron microscope (SEM). Using the SEM technique, we also easily confirmed the uniformity and feasibility of large-scale replication. The surface morphology of plasma-treated FEP samples is shown in Figure 4 for samples treated with 3 W and exposure times of 40 and 120 s. The linear pattern is maintained, and the results confirm its uniformity.

**Figure 5:**
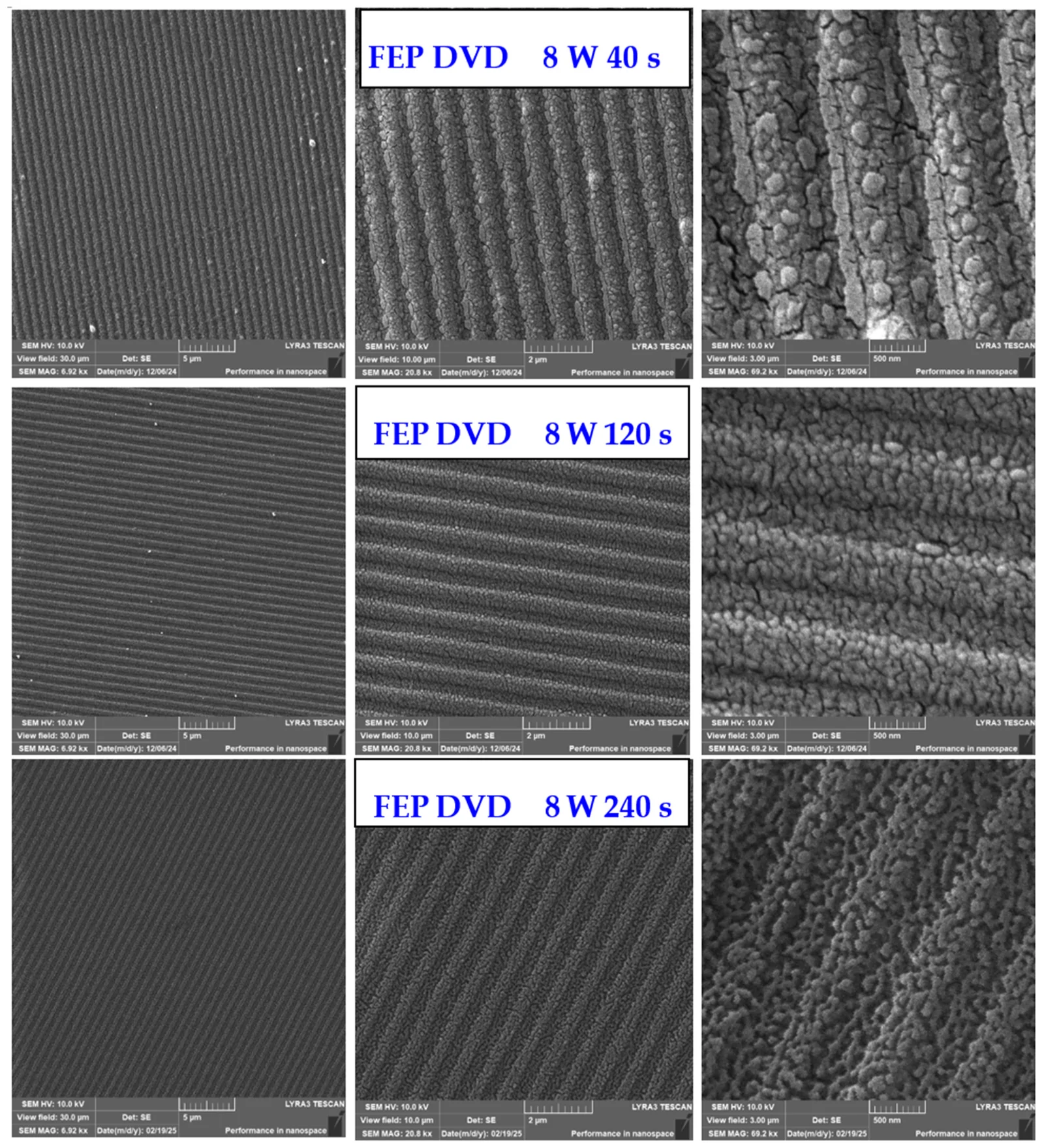
SEM images of DVD-replicated and plasma-modified FEP foils with 8 W power and plasma exposure times of 40 s and 120 s were chosen. Squares of 30 × 30 μm^2^ (the 1st column), 10 × 10 μm^2^ (the 2nd column) and 3 × 3 μm^2^ (the 3rd column) are shown.

The surface morphology of plasma-treated samples is shown in Figure 5. The samples treated with plasma power of 8 W and exposure times of 40 s, 120 s, and 240 s are introduced, and the different scanning areas are presented. The SEM scans confirmed detailed changes in surface morphology, including the appearance of worm-like structures due to plasma exposure, especially at longer exposure times, while the periodic pattern was maintained. The results are in good accordance with the AFM results. As is obvious, with large exposure times, the surface ablation induced a more pronounced superposed worm-like structure. This formation is associated with a different ablation ratio between the amorphous and crystalline phases, as described in detail in our earlier study [38].

The same setup was also used for energy-dispersive analysis (EDS), which provides elemental analysis information. The aim was to study the differences in physicochemical properties, specifically the surface chemistry induced by plasma activation. Our primary focus was on comparing low and high plasma power (3 W vs. 8 W), followed by comparing different exposure times. SEM images of the “pristine” and plasma-treated FEP are shown as Supplementary Figure S1; the scans 3 × 3 μm^2^ and 10 × 10 μm^2^ are provided.

**Figure 6:**
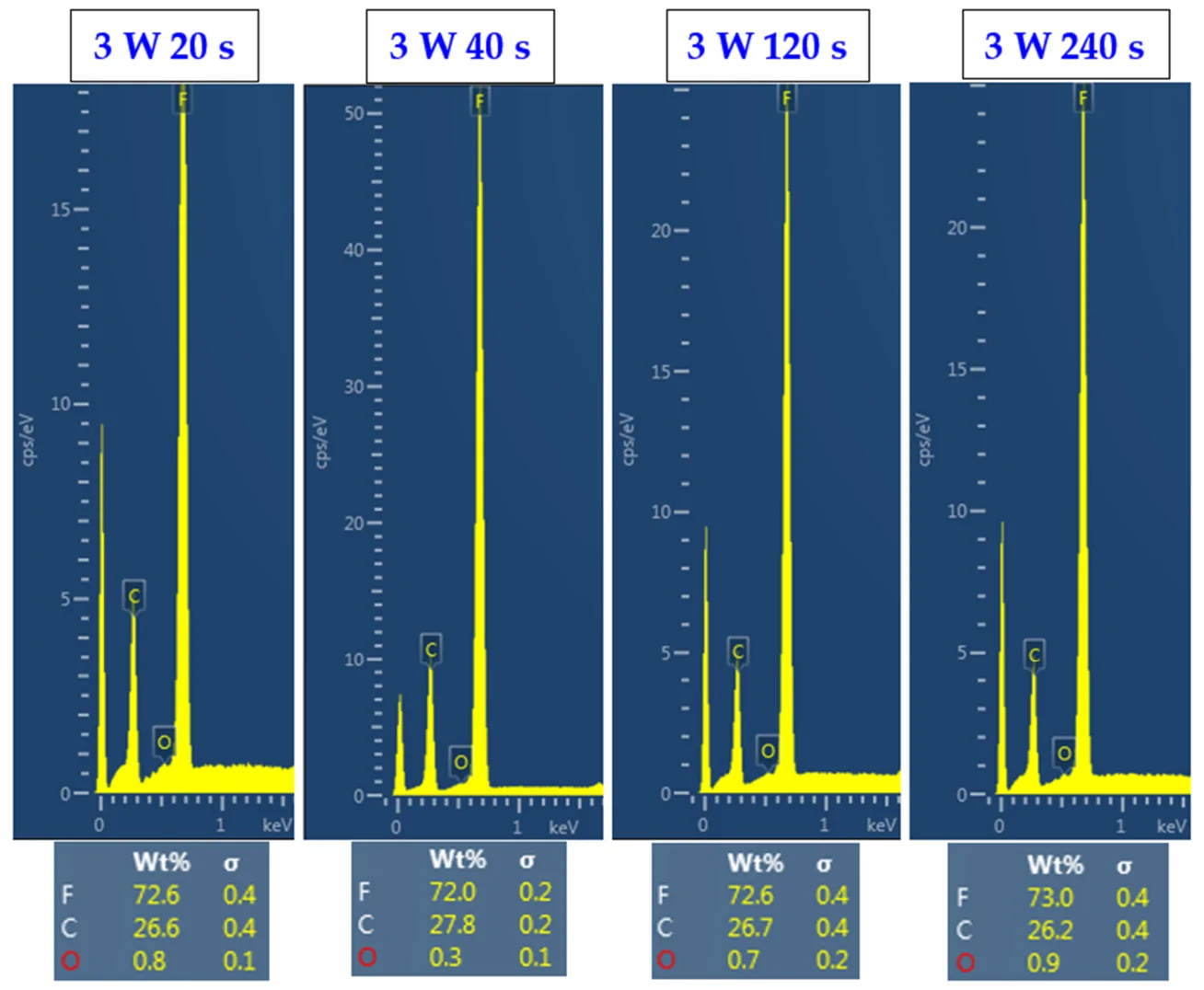
EDS spectra of DVD-replicated and plasma-modified FEP foil with 3 W power and plasma exposure times ranging from 20 s to 240 s were chosen. Representative elemental concentrations of fluorine, carbon, and oxygen are also introduced in wt.%.

The comparison of different exposure times (20–240 s) to study changes in oxygen concentration is shown in Figure 6. These EDS spectra clearly demonstrate that the plasma activation led to the incorporation of oxygen-containing groups onto plasma-activated FEP; this effect has been described previously [38,39].

A very surprising result was revealed, especially for higher exposure times. We expected a continual increase in oxygen surface concentration; however, only a slight increase in oxygen concentration was detected, and in some cases, even a decrease was detected (3 W and 20 s vs. 40 s). This surprising fact can be explained by two key factors. The first factor is based on the principle of argon plasma treatment, which affects only the very surface of the exposed polymer. As a result, the EDS technique, which allows us to acquire information at significantly greater depth (up to hundreds of nanometers), detects surface oxygen. However, compared to the remaining bulk material, which is unaffected by the Ar plasma, the percentage is small.

**Figure 7:**
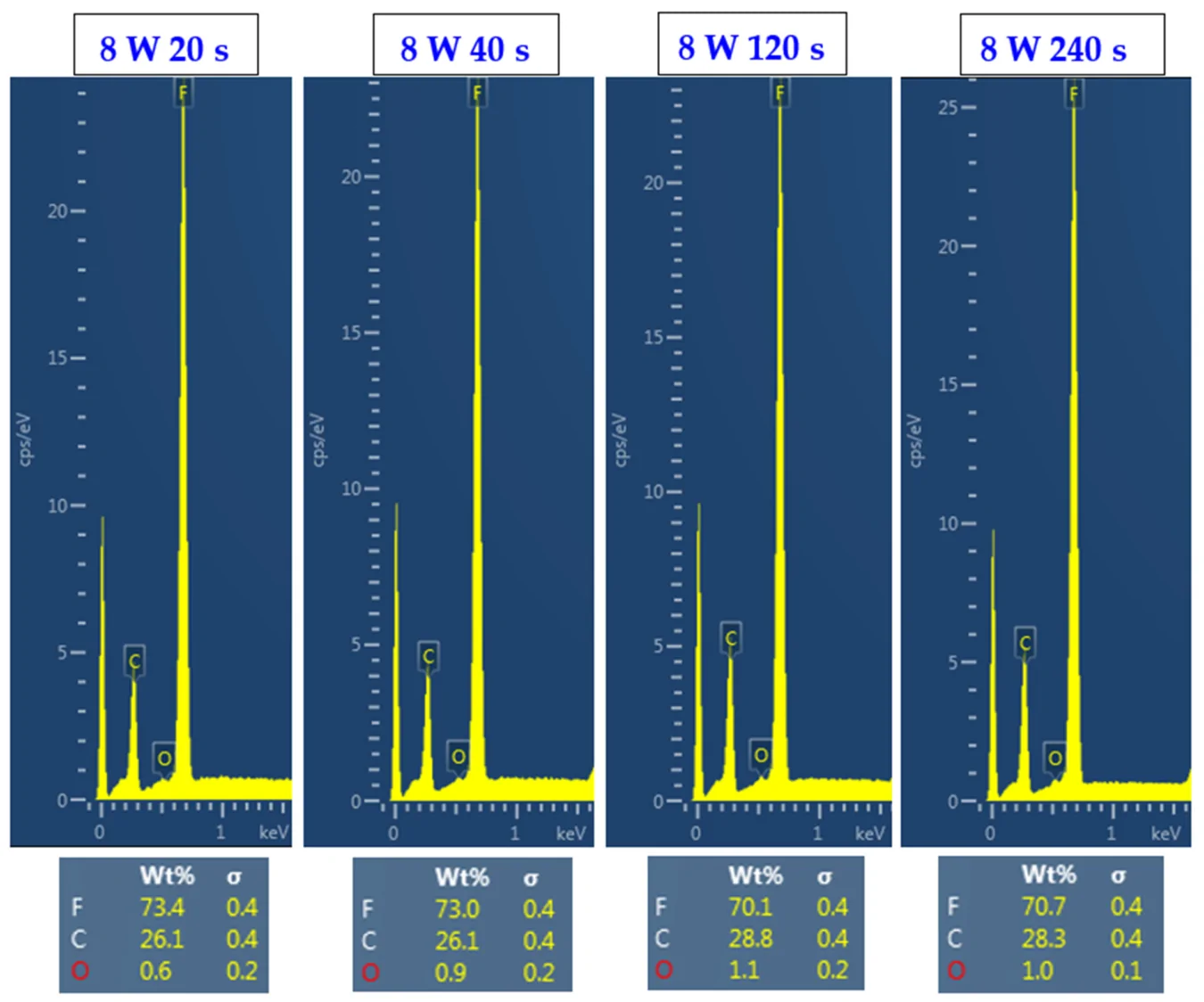
EDS spectra of DVD-replicated and plasma-modified FEP foils with 8 W power and plasma exposure times ranging from 20 s to 240 s were chosen. Representative elemental concentrations of fluorine, carbon, and oxygen are also introduced in wt.%.

The second factor is based on the plasma exposure time. As the plasma not only activates the surface but also induces material removal via ablation, a decrease in oxygen concentration may occur with higher exposure. The exposure at a plasma power of 8 W for exposure times ranging from 20 s to 240 s is shown in Figure 7. It is obvious that the increase in exposure time is related to the increase in oxygen concentration; however, for maximum exposure time, there is no significant change compared to 120 s. Also, when we compare different plasma powers, an increase in oxygen concentration is apparent but not significant. Therefore, we proceeded with a more specific type of surface elemental analysis, i.e., X-ray photoelectron spectroscopy (XPS), which provides information on surface physicochemical changes at the very top of the surface.

### 3.3. X-Ray Photoelectron Spectroscopy

**Figure 8:**
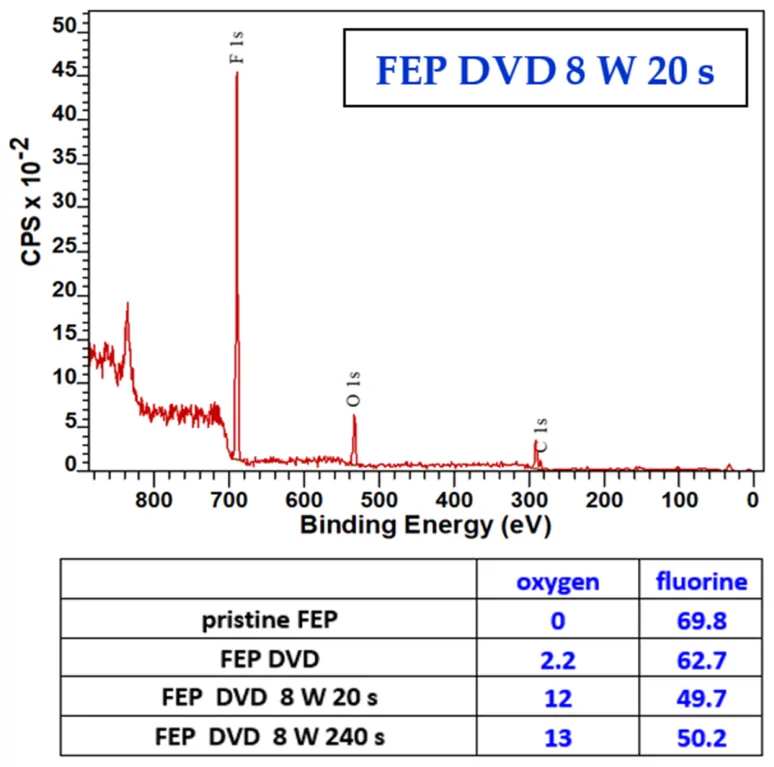
Representative XPS spectrum of DVD-replicated and plasma-modified FEP foil with 8 W power and a plasma exposure time of 20 s. In the table, the oxygen and fluorine concentrations for pristine FEP, pristine FEP with a replicated DVD pattern (FEP DVD), and this sample subsequently exposed to a plasma power of 8 W for 20 s and 240 s are presented. Representative elemental concentrations of fluorine and oxygen are presented in at.%.

As suggested in the previous paragraph, the changes to the ripple-replicated FEP pattern were predominantly induced on the top surface layer. Therefore, we analyzed the treated surfaces using XPS, a technique that provides information on approximately the top 10 atomic layers. The results of the oxygen and fluorine concentration analysis are presented in Figure 8.

As expected, the pristine FEP substrate, which was analyzed for comparison, showed no surface oxygen. A similar result could also be expected for pristine FEP, which was structured by the replication process (FEP DVD). However, as shown in Figure 8, after the replication process, the oxygen content increases slightly to 2.2 at.%. Since only a very thin surface layer was affected, the EDS did not reveal any significant amount for this sample; however, XPS did detect a change. Plasma treatment had a substantial effect on the grooved FEP substrate; even the shortest exposure time of 20 s significantly increased the atomic concentration up to 12 at.%. Not all the studied samples are presented here, as we wanted to demonstrate the major differences between pristine FEP and the selected types of modification. Further increasing the plasma exposure time had no significant influence on the oxygen concentration; for 240 s and 8 W, we detected 13 at.% of oxygen. However, the morphology of the samples changed substantially, as shown in the chapter related to AFM analysis [43,44].

We have also measured the wettability of the modified and replicated patterns. The contact angle of pristine FEP is 106°, indicating hydrophobicity. The value was also determined for the replicated pristine FEP with the pattern. We conclude that the patterning of the linear structure did not reduce the contact angle. Surprisingly, the plasma treatment did not affect the surface wettability of FEP as much as in the previous cases for pristine FEP without a pattern [38,39]; even a significant decrease was observed. In the case of plasma-treated non-patterned FEP, the changes of one of the physico-chemical properties, the wettability, were connected only to the changes in surface chemistry, while the surface morphology changes were determined to be on a nanometre-scale level, and a worm-like pattern appeared. A similar change was observed for patterned FEP in this work; the worm-like pattern appeared after plasma exposure. However, the original replicated submicron pattern was maintained, which was probably the main reason for the difference in the determined wettability. The plasma treatment at 8 W for 20 s reduced the contact angle to 88° ± 3.4°. Further increase of plasma exposure time under the same plasma power led to a decrease of contact angle to 85° ± 2.9° for exposure time 40 s, 83.2° ± 4.0° for exposure time 80 s, 81.4° ± 4.2° for exposure time 120 s, and 80.8° ± 7.4° for exposure time 240 s. It is evident that a further increase in exposure time led to a slight decrease in contact angle; the replicated pattern probably stabilized the surface contact angle.

### 3.4. Zeta-Potential Analysis

One of the key elements related to changes in surface chemistry is zeta-potential determination. Therefore, we analyzed the pristine and plasma-treated samples to determine their zeta-potential, as this is an important factor influencing cytocompatibility [45].

**Figure 9:**
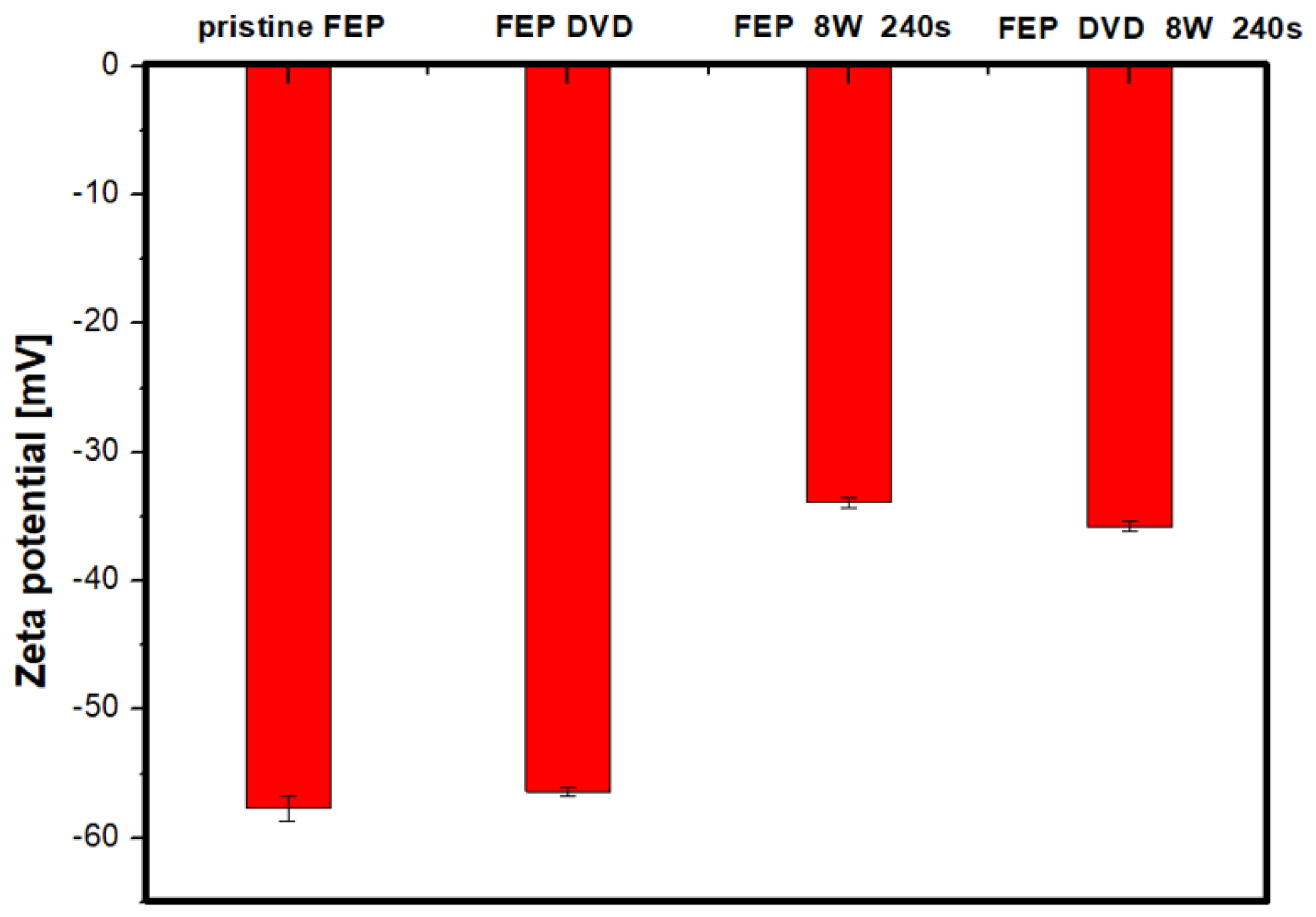
Zeta-potential (method HS) of pristine FEP, pristine FEP with a replicated DVD pattern (FEP DVD), and this sample subsequently exposed to plasma power of 8 W for exposure times of 20 s and 240 s.

As shown in Figure 9, the synergistic effect of changes in surface chemistry and morphology dramatically modified the zeta potential. Plasma exposure dramatically increased the zeta potential of both pristine and nanostructured FEP compared to pristine FEP and pristine FEP with a replicated submicron-scale pattern, making the surfaces more hydrophilic, consistent with the contact angle measurements mentioned above and with an increased oxygen content revealed by XPS. There is minimal difference between replicated FEP subsequently treated with plasma and plasma-treated FEP lacking a replicated submicron-scale pattern, suggesting that the plasma exposure is homogeneous and affects the entire area of the pattern similarly. These findings further suggest that the surface morphology itself does not affect the zeta-potential.

### 3.5. Cytocompatibility

The interaction between polymer matrices and human cells determines the material’s cytocompatibility. For these tests, we have chosen the samples treated with 8 W plasma for 240 s, based on our previously acquired data on plasma modification of FEP and its interaction with cells [39]. Mesenchymal stromal cells (MSCs) derived from Wharton’s jelly of the human umbilical cord were selected as a model for evaluating the cytocompatibility of the investigated polymer matrices. In our previous study, we confirmed that hWJSCs have a high proliferative capacity, which they maintain into higher passages, and their average population doubling time is shorter than 24 h [32]. Moreover, these cells are derived from the umbilical cord, which is usually discarded as waste material after delivery, and their collection is not burdened by ethical issues [46,47]. Once frozen, these cells serve as a readily available source of MSCs. Interestingly, WJSCs are considered less mature than MSCs derived from adult tissues, i.e., they are at the border between multipotency and pluripotency, express some markers of pluripotency, and have the potential to differentiate into different cell types [47]. Similarly, due to its favorable mechanical properties, thermal stability, chemical inertness, transparency, and gas (oxygen) permeability, FEP is promising for a wide range of biomedical applications, including modern approaches to cell cultivation for diagnosis and cell therapy [29,30,48], microfluidics and organ-on-chip strategies [49], advanced microscopy and label-free imaging techniques [50], and stent coating [51]. Therefore, the combination of WJSCs and FEP shows great promise for versatile applications in tissue engineering.

However, in its pristine unmodified state, FEP is hydrophobic and rather bioinert, i.e., not promoting a proper adsorption of cell adhesion-mediating proteins (e.g., vitronectin and fibronectin) from cell culture media, i.e., in a geometrical conformation suitable for binding the cell adhesion receptors, and the subsequent cell adhesion and growth [30,52]. In addition, hydrophobic surfaces preferentially adsorb serum albumin, which is non-adhesive for cells (for a review, see [53]). Therefore, we attempted to increase its bioactivity through morphological and physicochemical surface modifications, such as surface patterning via DVD replication and/or plasma treatment. Our earlier studies [38,39,41], and studies by other authors [30,52] have proven that these types of surface modification, namely the creation of a honeycomb-like pattern on FEP and/or plasma modification of this polymer, are an efficient tool for enhancing the adhesion, growth and differentiation of various cell types, such as vascular endothelial cells [52], epidermal keratinocytes [39], adipose-derived mesenchymal stem cells, dermal fibroblasts [38], lung fibroblasts and osteoblast-like cells [41].

**Figure 10:**
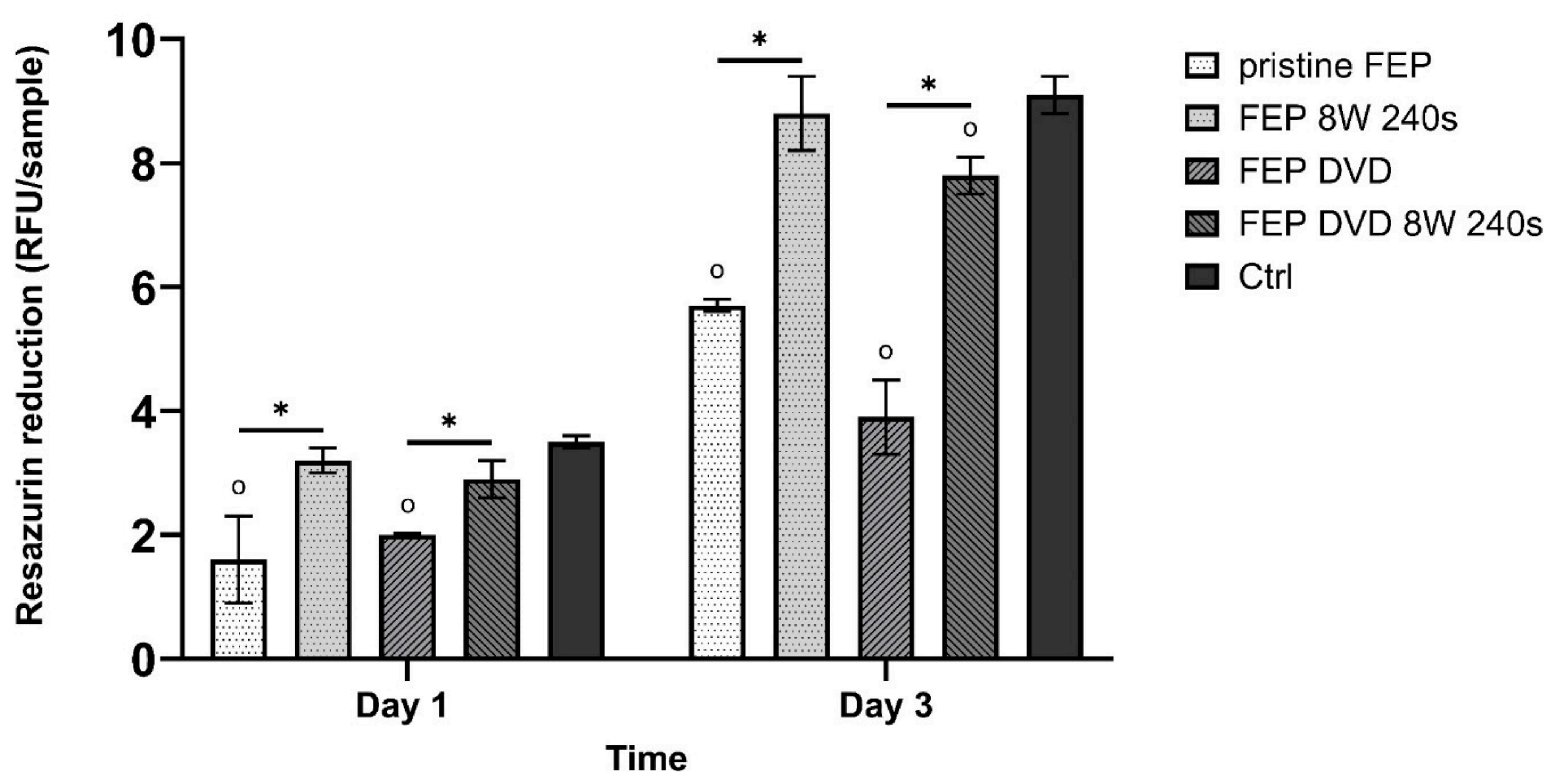
Metabolic activity of human Wharton’s jelly-derived MSCs growing on differentially treated FEP substrates and control tissue culture polystyrene (Ctrl), determined by resazurin assay at 1 and 3 days post-seeding. Data are represented as Mean ± SD. (°) indicates a statistically significant difference (*p* < 0.05) compared to the control group; (*) indicates a statistically significant difference (*p* < 0.05) between untreated and plasma-treated FEP samples (*n* = 6) in the same time interval. One-way ANOVA, Student–Newman–Keuls method, *p* ≤ 0.05.

However, as shown in Figure 10, there was no statistically significant difference in metabolic activity between cells cultured on pristine unmodified FEP and DVD-replicated FEP discs at either 24 or 72 h after cell plating. After 72 h, the metabolic activity on DVD-replicated FEP discs even tended to be lower than that on pristine unmodified FEP, though this difference was not statistically significant. Meanwhile, the metabolic activity of cells on DVD-replicated FEP discs at both time points was significantly lower than that of the control cells grown on tissue culture polystyrene. This result was rather surprising because we had expected an increase in cell attachment, growth, and alignment in the direction of the grooves on the material surface, as observed in our previous studies on microgrooved polydimethylsiloxane (PDMS) and nanogrooved polyether ether ketone (PEEK) and polyethylene naphthalate (PEN) [34,35]. However, in the case of PDMS, the surface pattern was activated by plasma and coated with collagen—a protein that promotes cell adhesion—whereas for PEEK and PEN, laser treatment was used to pattern the surface, increasing its wettability, which is advantageous for cell adhesion and growth. In our present study, however, FEP modified only by DVD replication remained hydrophobic, with low oxygen content and low zeta potential, i.e., factors associated with low material attractiveness for cell adhesion (for a review, see [53]). In addition, on PDMS, the grooves were wider, 10–100 micrometers in width, which better corresponded to the size of the cell adhesion area than the grooves on FEP in our present study, which were only approximately 0.5 µm wide [34]. Cells adhere to areas on the order of tens of micrometers, and submicrometer-scale grooves might be too narrow for them.

**Figure 11:**
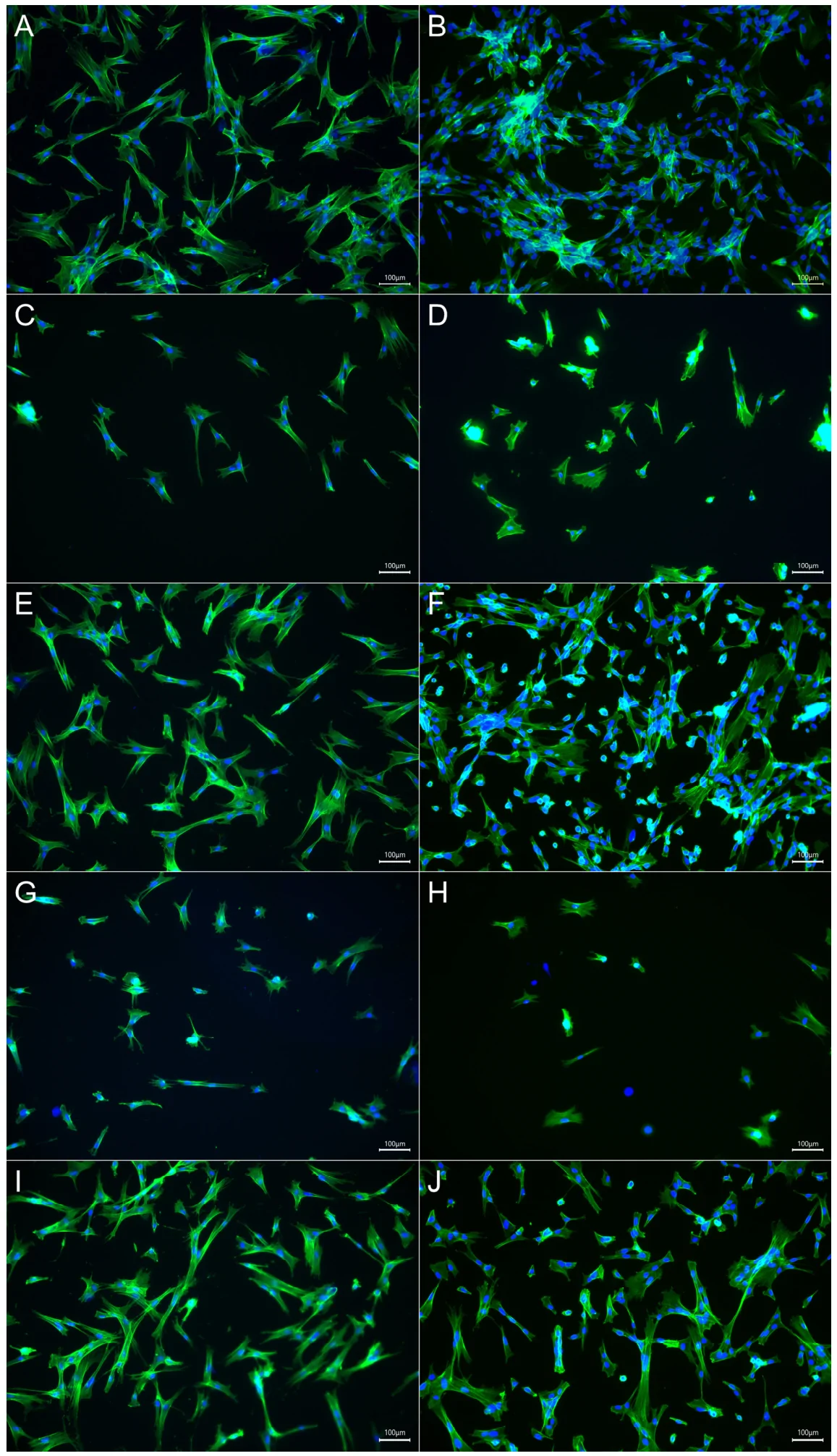
Fluorescence microscopy images of human Wharton’s jelly-derived MSCs on day 1 (left column) and day 3 (right column) post-seeding on control tissue culture polystyrene (**A**,**B**), pristine FEP (**C**,**D**), FEP treated only by plasma 8 W 240 s (**E**,**F**), FEP treated only by DVD replication (**G**,**H**), and FEP treated by both DVD-replication and plasma 8 W 240 s (**I**,**J**). Cell nuclei stained with Hoechst (blue), F-actin labeled with phalloidin-Atto 488 (green). The scale bar = 100 μm.

However, after plasma treatment, both pristine and DVD-replicated FEP became markedly more attractive for cell attachment. Cell metabolic activity on these substrates increased significantly, reaching levels similar to those on the control tissue culture polystyrene, i.e., another plasma-treated polymer (Figure 10). The difference between the pristine unmodified and plasma-treated samples became even more pronounced 72 h after cell seeding. At this time point, the metabolic activity of cells on plasma-treated FEP discs was 54.4% higher than on pristine FEP discs, and on plasma-treated DVD-replicated FEP discs, it was even 100% higher than on corresponding DVD-replicated-only discs. These results suggest that DVD replication made the FEP samples more susceptible to subsequent plasma modification, probably due to their larger effective surface area. On the other hand, the cell metabolic activity on plasma-treated DVD-replicated FEP discs still remained lower than on control standard tissue culture polystyrene, while the value on plasma-treated non-patterned FEP samples was comparable to that for polystyrene. One possible explanation is that the DVD replication produces submicron-scale irregularities, such as grooves and prominences approximately 0.5 µm wide, on the material surface. As mentioned above, the cells typically spread over tens of micrometers, and micron- and submicron-scale irregularities could impede cell spreading; for a review, see [53,54]. In addition, as shown in Figure 11, the cells remained randomly distributed on the material surface, without alignment with the pattern. The cell guidance by patterned materials could be improved by modulating the groove size from submicron to tens of micrometers to better match cell size. Alternatively, the pattern could be further functionalized with cell adhesion-mediating proteins such as collagen [34], vitronectin and fibronectin [52], or adhesion oligopeptides derived from these proteins [55], preferably by alternating strips with these biomolecules and strips without them [56].

Metabolic activity was determined by a resazurin assay, and the data are presented as the mean relative fluorescence units (RFU) ± standard deviation for each sample. The resazurin assay is a method used to evaluate cell viability and material cytotoxicity. It works by measuring cell metabolic activity, assessed by the conversion of the non-fluorescent dye resazurin to the fluorescent dye resorufin. This conversion is caused by mitochondrial enzymes in proliferating living cells. The resulting fluorescence intensity is therefore correlated with the number of viable cells and can be considered an indirect marker of cell proliferation activity [31,33].

For further evaluation of the cells’ response to FEP samples, cell behavior (morphology and attachment to the tested substrates) was monitored using a fluorescence microscope (Figure 11) by labeling F-actin, which visualized stress fibers. After the first day, elongated spindle-shaped cells with a morphology characteristic of MSCs could be observed in all tested and control samples. In all tested samples, the cells had a shape similar to that of the control group.

However, cells growing on plasma-untreated FEP discs without and with DVD-replication) were less spread out than cells growing on plasma-treated FEP discs and on control tissue culture polystyrene. In addition, the cells in all groups were distributed randomly without any apparent directionality, i.e., alignment along the grooves. At both time points, an increase in cell population density was observed on plasma-treated FEP discs compared to untreated FEP discs. This was particularly apparent on day 3, when the cells on plasma-treated discs reached densities of 25,428 ± 702 and 59,909 ± 2665 cells/cm^2^ on DVD-replicated and non-replicated discs, respectively, whereas on plasma-untreated discs, the densities were only 3831 ± 991 and 7755 ± 771 cells/cm^2^. These results are consistent with cell metabolic activity and indicate that the plasma treatment has a major positive effect on cell performance rather than on DVD replication.

## 4. Conclusions

We confirmed successful replication of FEP foils and the maintenance of a regular linear pattern; the linear pattern was successfully transferred into FEP polymer. The brief treatment at lower plasma power did not significantly affect the surface, and the detailed surface morphology further confirmed the maintenance of the ripple pattern. The plasma treatment induced significant chemical changes on the replicated FEP surface; the wrinkle-like structure was superimposed on the linear LIPSS-like pattern of the replicated FEP, and the effective surface area was substantially enhanced. The changes in surface morphology of replicated and/or plasma-treated patterns were confirmed with SEM analysis. EDS analysis revealed that plasma activation incorporated oxygen-containing groups into the plasma-activated FEP; however, this treatment affected only the very surface of the exposed polymer. Plasma treatment has a substantial effect on the grooved FEP substrate, as detected by XPS; even at the shortest exposure time of 20 s, the atomic concentration increases significantly, up to 12 at.%. Further increases in plasma exposure time did not significantly affect the oxygen concentration, and at 240 s and 8 W, we detected 13 at.% of oxygen, but the morphology of the samples is changed substantially, as was shown in the chapter related to AFM analysis. The synergistic effect of surface chemistry and morphology changes dramatically altered the zeta potential; plasma exposure of both pristine and replicated FEP markedly increased the zeta potential of the surface compared to pristine FEP and pristine FEP with a replicated pattern, thereby making the surface more hydrophilic. Cultivation of human Wharton’s jelly mesenchymal stromal cells revealed that FEP samples modified by replication and/or plasma treatment supported cell adhesion, spreading, metabolic activity, and proliferation. This positive effect was mainly due to plasma modification; however, the replication patterning of the FEP surface likely increased its sensitivity to plasma treatment, as suggested by a more pronounced increase in cell metabolic activity on patterned plasma-modified surfaces.

## Figures and Tables

**Table 1:** Samples used for the cytocompatibility study and their abbreviations used throughout the manuscript.

Sample Characterization	Sample Abbreviation in the Manuscript
pristine unmodified FEP	pristine FEP
pristine FEP modified with plasma	FEP 8 W 240 s
FEP replicated with DVD pattern	FEP DVD
FEP modified by both DVD replication and plasma exposure	FEP DVD 8 W 240 s

## Data Availability

Data are available at the link https://doi.org/10.5281/zenodo.17522312.

## Supplementary Materials

The following supporting information can be downloaded at: https://www.mdpi.com/article/10.3390/jfb17080364/s1, Figure S1: SEM images of pristine (left column) and plasma-modified FEP foil with 8 W power and plasma exposure time 240 s (right column) are introduced. Squares of 10 × 10 μm^2^ and 3 × 3 μm^2^ are shown.
