# Supplementary material for: Plasma Treatment Enhances Cytocompatibility on Patterned Perfluoroethylene Propylene
Source: J Funct Biomater. 2026 Jul 28;17(8):364. doi: 10.3390/jfb17080364 (PMC13514769; doi:10.3390/jfb17080364)
Supplement: Supplementary file 1 [file jfb-17-00364-s001.zip › jfb-4388760-supplementary.pdf]

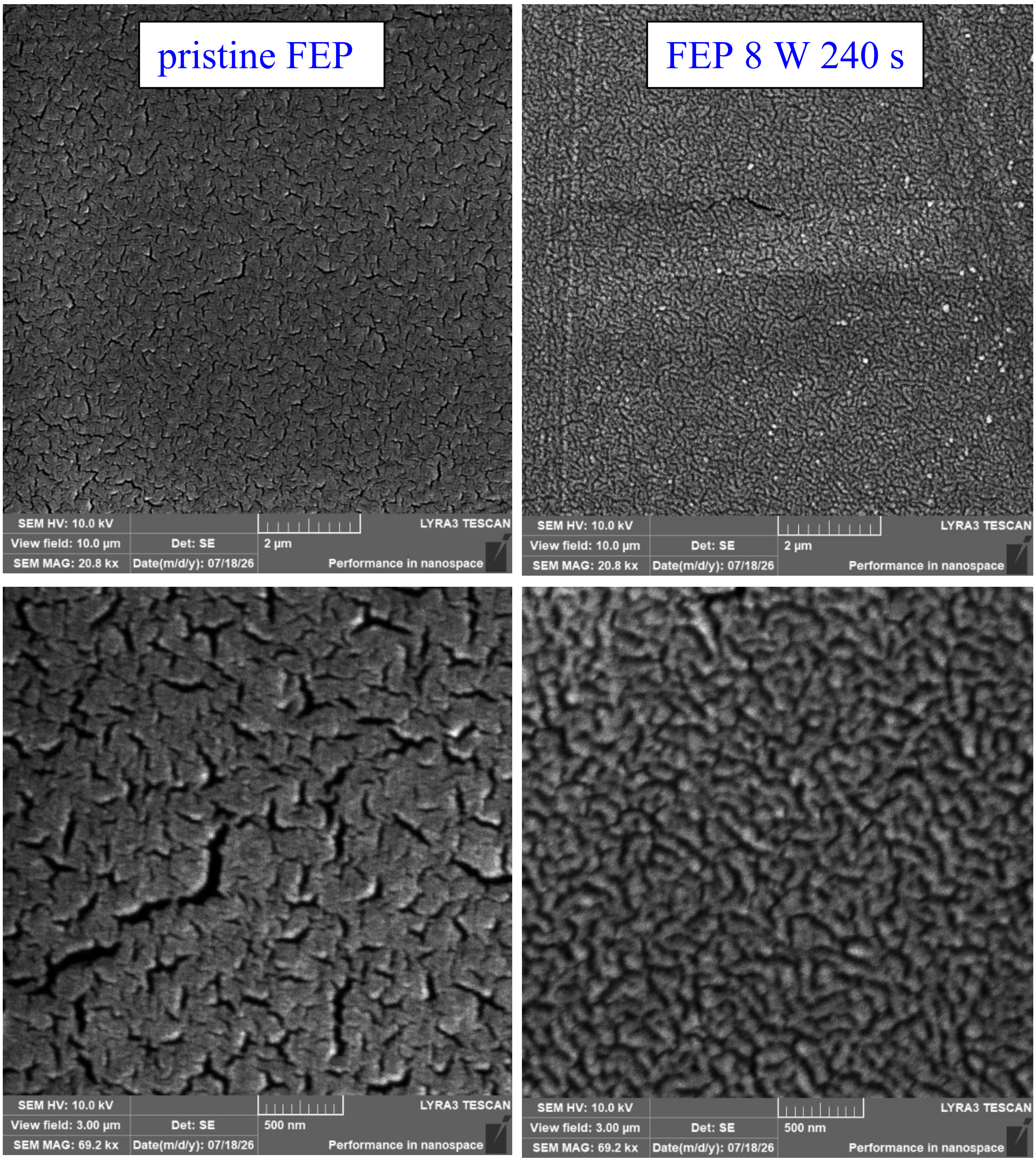

Figure S1. SEM images of pristine (left column) and plasma-modified FEP foil with 8 W power and plasma exposure time 240 s (right column) are introduced. Squares of 10 × 10 µm^2 and 3 × 3 µm^2 are shown.
